# A primary pediatric acute myelomonocytic leukemia with t(3;21)(q26;q22): A case report

**DOI:** 10.1097/MD.0000000000035721

**Published:** 2023-10-27

**Authors:** Jia-xin Duan, Fang Liu, Li Chang, Guang-lu Che, Qiu-xia Yang, Jie Teng, Hui Jian, Xiao-juan Liu, Shu-yu Lai

**Affiliations:** a Department of Laboratory Medicine, West China Second University Hospital, and Key Laboratory of Birth Defects and Related Diseases of Women and Children (Sichuan University), Ministry of Education, Sichuan University, Chengdu, China.

**Keywords:** case report, *MECOM*, *RPL22*, *RUNX1*, t(3;21)

## Abstract

**Rationale:**

The rare t(3;21)(q26;q22) translocation results in gene fusion and generates multiple fusion transcripts, which are typically associated with therapy-related myelodysplastic syndrome, acute myeloid leukemia, and chronic myelogenous leukemia. Here, we report a rare case of de novo acute myelomonocytic leukemia in a young child with t(3;21)(q26;q22).

**Patient concerns:**

A 2-and-a-half-year-old female patient presented with abdominal pain, cough, paleness, and fever for 3 weeks, without any history of malignant diseases.

**Diagnoses:**

Chest computed tomography revealed pneumonia. Bone marrow smear confirmed acute myelomonocytic leukemia. Cytogenetic analysis and Sanger sequencing identified *RUNX1-MECOM* and *RUNX1-RPL22* fusion genes as a result of t(3;21)(q26;q22).

**Interventions:**

The patient received 3 courses of chemotherapy, but bone marrow smear examination showed no remission. According to the wishes of the patient family, the allogeneic hematopoietic stem cell transplantation (Allo-HSCT) was chosen.

**Outcomes:**

The patient did not experience any adverse reactions after Allo-HSCT. The red blood cells and platelets increased without transfusion. The pneumonia recovered after antibiotic treatment.

**Lessons:**

The patient recovered well after Allo-HSCT. Therefore, for patients with *RUNX1-MECOM* and *RUNX1-RPL22* fusion genes, transplantation may be a good choice when chemotherapy is not effective.

## 1. Introduction

The translocation of t(3;21)(q26;q22) is a rare cytogenetic abnormality whose molecular mechanisms have been elucidated due to the development of sequencing. This translocation results in the fusion of the *RUNX1* (also called *AML1*) gene at 21q22 with the *MECOM* (also called *MDS1-EVI1*) gene and the *RPL22* (also called *EAP*) gene at 3q26, generating multiple fusion transcripts. Researches have shown that the appearance of this chromosomal abnormality was often associated with therapy-related myelodysplastic syndrome, therapy-related acute myeloid leukemia,^[1,2]^ the blastic crisis phase of chronic myelogenous leukemia,^[1,3,4]^ and on rare occasions, de novo AML.^[5]^ We here report the clinical manifestations, laboratory findings, and therapeutic outcome of a little girl diagnosed as de novo acute myelomonocytic leukemia (AML-M4) with *RUNX1-MECOM* and *RUNX1-RPL22* fusion genes.

## 2. Case presentation

A 2-and-a-half-year-old female patient was admitted to the Department of Hematology of West China Second University Hospital in Southwest China because of abdominal pain, cough, paleness, and fever. This child presented with a characteristic clinical picture of acute onset, with a course of 3 weeks at admission. The physical examination was normal, except for enlarged tonsils and hepatomegaly, no rash or petechiae were noted. There was no family or personal history of malignant diseases.

### 2.1. Investigations

**Figure 1.**
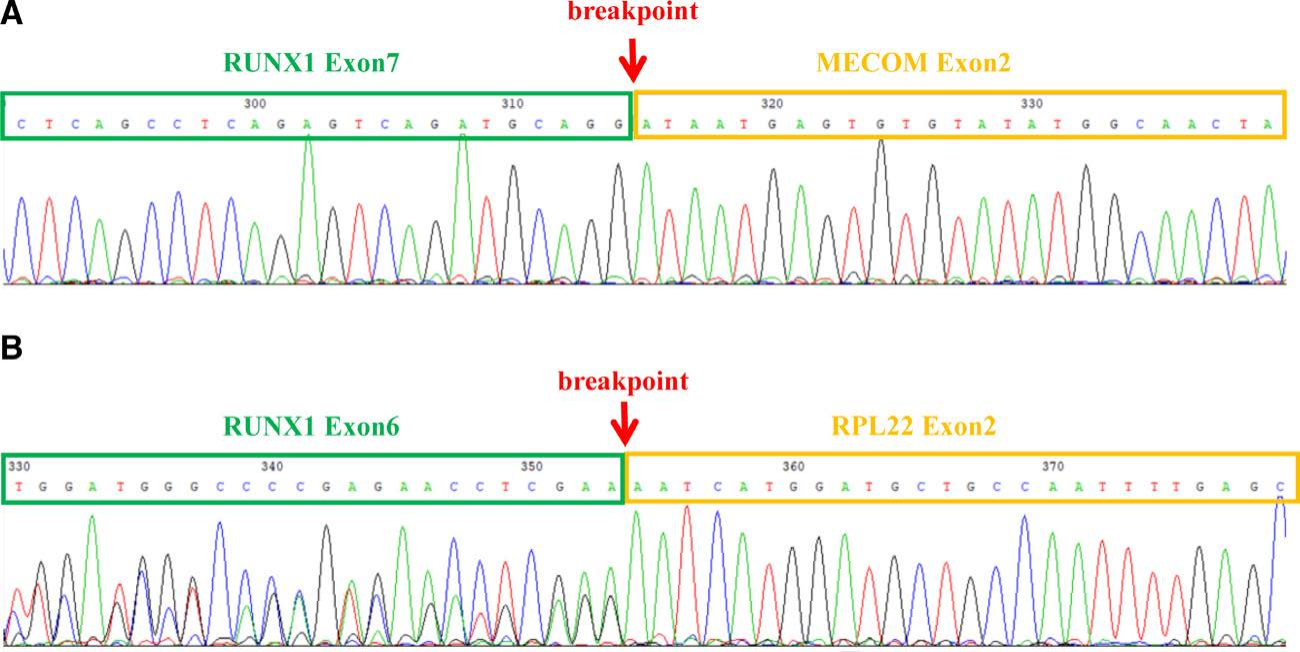
Sanger sequencing analyses revealed (A) *RUNX1-MECOM*, and (B) *RUNX1-RPL22* fusion transcripts, respectively.

Chest computed tomography showed lung inflammation, mainly in the right upper lobe. Ultra-sound radiography revealed multiple enlarged mesenteric lymph nodes. Blood routine examination showed a hemoglobin level of 65 g/L (reference range, 112–149 g/L), a white blood cell count of 22.4 × 10^9^/L (reference range, 4.4–11.9 × 10^9^/L), an absolute neutrophil count of 5.6 × 10^9^/L (reference range, 1.2–7.0 × 10^9^/L), a platelet (PLT) count of 25 × 10^9^/L (reference range, 125–462 × 10^9^/L), and a proportion of 31% juvenile cells. Bone marrow (BM) smear showed acute myelomonocytic leukemia type M4 (AML-M4). The BM immunophenotyping identified blast cells accounted for 52.29% of nucleated cells and were positive for CD13, CD14, CD15, CD33, CD34, and HLA-DR Cytogenetic analysis revealed an abnormal karyotype: 46, XX, t(3;21)(q26;q22) in all 20 analyzed metaphase cells. Molecular screening for 56 common fusion genes identified *RUNX1-MECOM* gene and *RUNX1-RPL22* gene, and the results were validated by Sanger sequencing (Fig. 1). Based on the above test results, the final diagnosis was AML-M4 with the coexistence of *RUNX1-MECOM* and *RUNX1-RPL22* fusion genes.

### 2.2. Treatment

**Figure 2.**
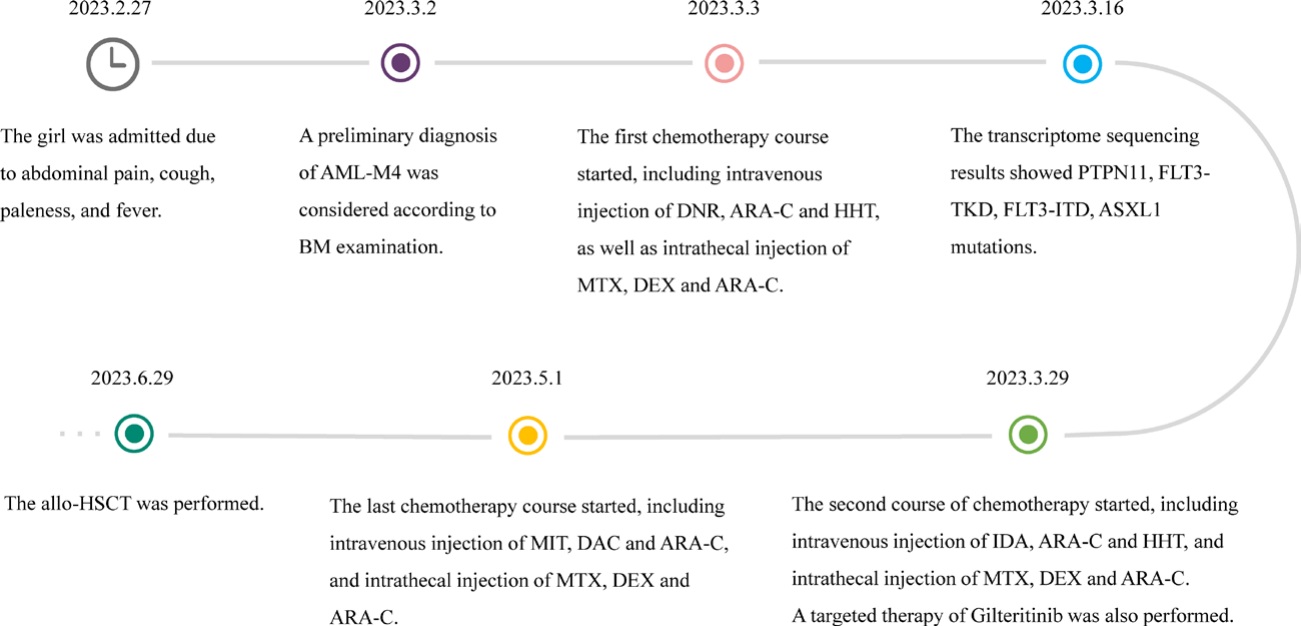
Timeline of the patient progress.

We have summarized the diagnostic and therapeutic process of this patient (Fig. 2). The first chemotherapy course started from March 3, 2023, including intravenous injection of daunorubicin, cytarabine (Ara-C) and homoharringtonine, as well as intrathecal injection of methotrexate (MTX, 10 mg), dexamethasone (DEX, 3 mg) and Ara-C (30 mg). BM smear displayed no remission of AML-M4 on the 21st day after the first chemotherapy course. BM minimal residual disease detected by multi-parameter flow cytometry showed that the primitive hematopoietic progenitor cells accounted for approximately 35.97% of the nucleated cells. During this period, the whole transcriptome sequencing for hematological malignancies results showed *PTPN11, FLT3-TKD, FLT3-ITD*, and *ASXL1* mutations. According to the presence of the FLT3 mutation, targeted therapy with gilteritinib was used since March 29. Meanwhile, the second course of chemotherapy began, including intravenous injection of idarubicin, Ara-C and homoharringtonine, as well as intrathecal injection of MTX (10 mg), DEX (3 mg) and Ara-C (30 mg). The last chemotherapy course started on May 1, including intravenous injection of mitoxantrone, decitabine and Ara-C, as well as intrathecal injection of MTX (10 mg), DEX (3 mg) and Ara-C (30 mg). The results of cerebrospinal fluid smear showed that no juvenile cells were found. Antibiotics were given to combat pneumonia, and recombinant human granulocyte colony-stimulating factor was injected to increase neutrophil count (the minimum value was 0.01 × 10^9^/L due to BM suppression during chemotherapy). In addition, red blood cells and platelets were transfused regularly according to blood routine examination results. After the completion of 3 chemotherapy courses, there were no signs of relief. The human leukocyte antigen (HLA) high-resolution revealed 7/12 match between the patient and her father, so the allogeneic hematopoietic stem cell transplantation (Allo-HSCT) was opted on June 29. There were no hyperacute or acute rejections after transplantation. On the 14th day after transplantation, the patient general condition was good, and there was currently no need to inject red blood cells or platelets. Blood routine examination showed a hemoglobin level of 83 g/L, white blood cell count of 2.2 × 10^9^/L, absolute neutrophil count of 1.63 × 10^9^/L, and PLT count of 40 × 10^9^/L. However, long-term follow-up is needed.

## 3. Discussion

The *RUNX1-MECOM* gene and *RUNX1-RPL22* gene are fusion transcription factors generated by t(3;21)(q26;q22) translocation. RUNX1 (AML1) is a DNA-binding subunit of the core binding factor, playing a pivotal role in hematopoiesis and blood vessel development.^[6]^ Studies have shown that *RUNX1-MECOM* fusion gene products can promote leukemogenesis by abrogating TGF-β-mediated inhibition of cell growth and stimulating proliferation.^[7,8]^ Mice transplanted with *AML1-MDS1-EVI1 (RUNX1-MECOM*)-transduced BM cells suffered from a disease similar to human AML after transplantation.^[9]^ RPL22 is an RNA binding component of the 60S ribosome subunit, which functions as a haplo insufficient tumor suppressor in T cell acute lymphoblastic leukemia (T-ALL).^[10,11]^ In both the T cell malignant tumor mouse models and in vitro acute transformation assays, it was found that the monoallelic loss of *RPL22* accelerated the development and dissemination of thymic lymphoma.^[10,11]^ The fusion of *RUNX1* and *RPL22* is not within the framework, resulting in the protein terminating shortly after fusion by introducing a stop codon, without tumor promoter characteristics in mouse experiments.^[12,13]^ However, the prognostic value of *RUNX1-RPL22* in human AML has not yet been determined. Mutations of *FLT3* are common genetic aberrations in newly diagnosed patients with AML,^[14]^ including *FLT3* internal tandem duplications (*FLT3*-ITD) and point mutations or deletions in the *FLT3* tyrosine kinase domain (*FLT3*-TKD), which are associated with poor prognosis.^[15]^ In this case, the patient harbored mutations in the *PTPN11 gene, ASXL1 gene, and RUNX1*-related fusion genes, all of which are frequent co-occurring genetic aberrations of *FLT3* mutation.^[16]^ A study has shown that *PTPN11* mutations appear to be associated with poorer survival and may confer resistance to gilteritinib.^[17]^ From the above information, it is clear that treating patients with those genetic aberrations is challenging. Acute myeloid leukemia with t(3;21)(q26.2;q22) is always associated with poor outcome.^[2]^ In this case, the patient did not respond satisfactorily to chemotherapy, indicating that traditional chemotherapy regimens may be ineffective for patients carrying *RUNX1-MECOM* and *RUNX1-RPL22* fusion genes.

Evidently, t(3;21)(q26;q22) is more common in adult patients with secondary diseases, most of whom had previously been treated with chemotherapy drugs or immunosuppressants.^[2,18]^ Our study reports a case of de novo AML without any special medical history. To the best of our knowledge, this child is the youngest patient of AML-M4 with t(3;21)(q26;q22), carrying both *RUNX1-MECOM* and *RUNX1-RPL22* fusion genes.^[5,19]^

## 4. Conclusion

In this case, the patient condition after transplantation was generally good, so Allo-HSCT might be a promising method for treating such patients when chemotherapy is not effective. However, due to the relatively short follow-up time, it is currently difficult to conclude that the patient has significantly benefited from transplantation. We will track the prognosis of the child and collect more similar cases to provide a basis for the diagnosis and treatment of such patients.

## Author contributions

**Conceptualization:** Jia-xin Duan, Shu-yu Lai.

**Data curation:** Li Chang, Guang-lu Che, Jie Teng.

**Funding acquisition:** Fang Liu, Xiao-juan Liu.

**Investigation:** Jia-xin Duan.

**Resources:** Fang Liu, Li Chang.

**Validation:** Qiu-xia Yang.

**Writing – original draft:** Jia-xin Duan.

**Writing – review & editing:** Hui Jian, Shu-yu Lai.
